# Chronic Fracture of the Posteromedial Tubercle of the Talus Masquerading as Os Trigonum Syndrome

**DOI:** 10.1155/2021/6637081

**Published:** 2021-06-28

**Authors:** Thananjeyen Srirangarajan, Ali Abbasian

**Affiliations:** Guy's & St Thomas' Hospital, London, UK

## Abstract

Posterior ankle impingement syndrome (PAIS) can be caused by osseous pathology from the posterior aspect of the talus. The commonest cause is an os trigonum, an accessory ossicle arising from the *lateral* tubercle of the posterior talus. We have observed cases where the osseous impingement is due to a chronic fracture nonunion of the *medial* tubercle of the posterior talus with unique symptoms, differentiating this clinical syndrome from the more common os trigonum syndrome. These can be readily overlooked on imaging and confused with an often coexisting os trigonum. Awareness of these lesions is paramount to ensure appropriate management and safe surgery. We describe a series of patients presenting to the senior author with this clinical syndrome, discuss its unique clinical and radiological features, and describe our surgical technique.

## 1. Introduction

Posterior ankle pain is a characteristic feature of posterior ankle impingement syndrome (PAIS). Pain can be exacerbated with plantarflexion and repetitive loading maneuvers of the ankle [1]. The posterior process of the talus is divided into the lateral tubercle and a smaller medial tubercle by the flexor hallucis longus (FHL) tendon groove. The posterolateral tubercle is described as Stieda's process and provides a bony attachment for the posterior talocalcaneal and talofibular ligaments [2, 3]. The posteromedial tubercle provides a bony attachment for the posterior third of the deltoid ligament superiorly and the medial talocalcaneal ligament inferiorly [2, 3]. Osseous pathology from the posterolateral tubercle of the talus is the commonest cause of PAIS [4] An os trigonum is an accessory ossicle that is an anatomical variant found in 25% of the population. It arises from the failed secondary ossification of the posterolateral tubercle of the talus [5].

We present four cases presenting with posteromedial ankle pain; all patients had become aware of the appearance of a painful bony protrusion on the medial aspect of the posterior ankle with symptoms of posterior impingent. In some cases, there was no report of a single traumatic episode preceding the onset of symptoms; however, all four patients were able to recall recurrent ankle injuries whilst playing football (soccer) in the past. In all cases, imaging revealed fragmentation and nonunion of a fracture to the medial tubercle of the talus with secondary features of posterior ankle impingent. Plain lateral projection radiographs had shown all patients to have a coexisting os trigonum. Investigative radiography reports had difficulty distinguishing the os trigonum from the talus posteromedial tubercle fracture nonunion. Without an index of suspicion, this can lead to the misdiagnosis of os trigonum syndrome and thus lead to the failure of surgical treatment. The aim of this paper is to raise awareness of the rare variant in cases of PAIS.

## 2. Cases

### 2.1. Case 1

**Figure 1:**
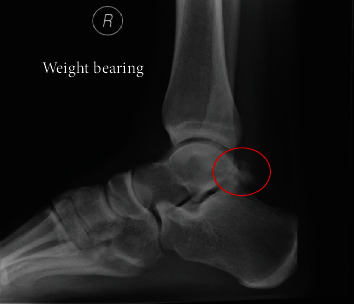
Lateral radiograph of right ankle showing an os trigonum and posterior talus process fracture.

**Figure 2:**
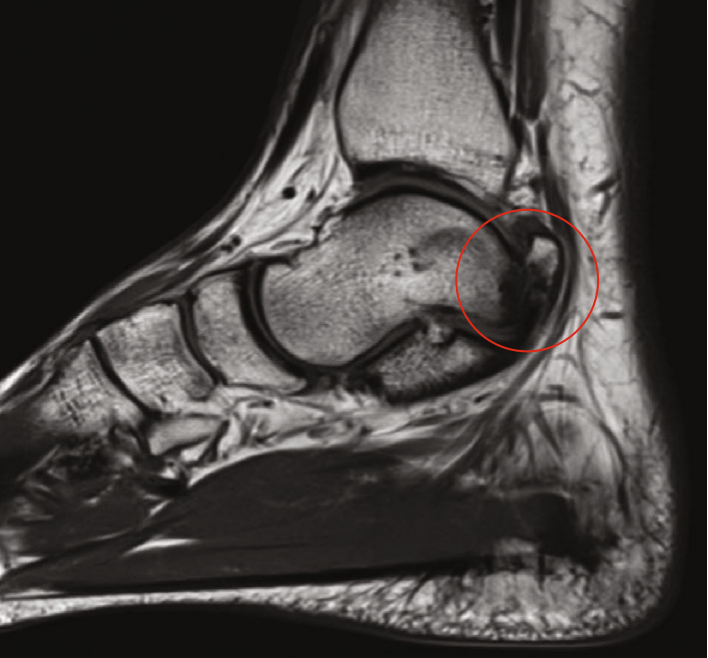
Sagittal slice of T2 MRI of right ankle showing the posteromedial talus fracture fragment and associated oedema.

A 25-year-old physiotherapist, who had previous trouble with anterior ankle impingement that was treated with arthroscopic surgery, presented with PAIS. He was a recreational football (soccer) player but did not report any single significant sporting injuries. He described chronic pain and had noted a bony swelling in the posteromedial aspect of his ankle. The plain radiograph of the ankle demonstrated an os trigonum; however, due to the orientation of the medial tubercle, this was not clearly seen and he was incorrectly diagnosed as having os trigonum syndrome (see Figure 1). The MRI scan reported mild inflammation associated with the os trigonum but also confirmed the presence of bone fragments posteromedially between FHL and the flexor digitorum longus (FDL) with marrow oedema affecting the medial tubercle of the talus (see Figure 2).

After failure of conservative treatment, he underwent surgery. Due to the position of the medial bony fragments, an open rather than an endoscopic excision was performed to avoid injury to the posterior tibial neurovascular bundle. The os trigonum was felt not to be contributary to the symptoms and was left alone. Surgery was uneventful and at final review reported resolution of the preoperative symptoms.

### 2.2. Case 2

**Figure 3:**
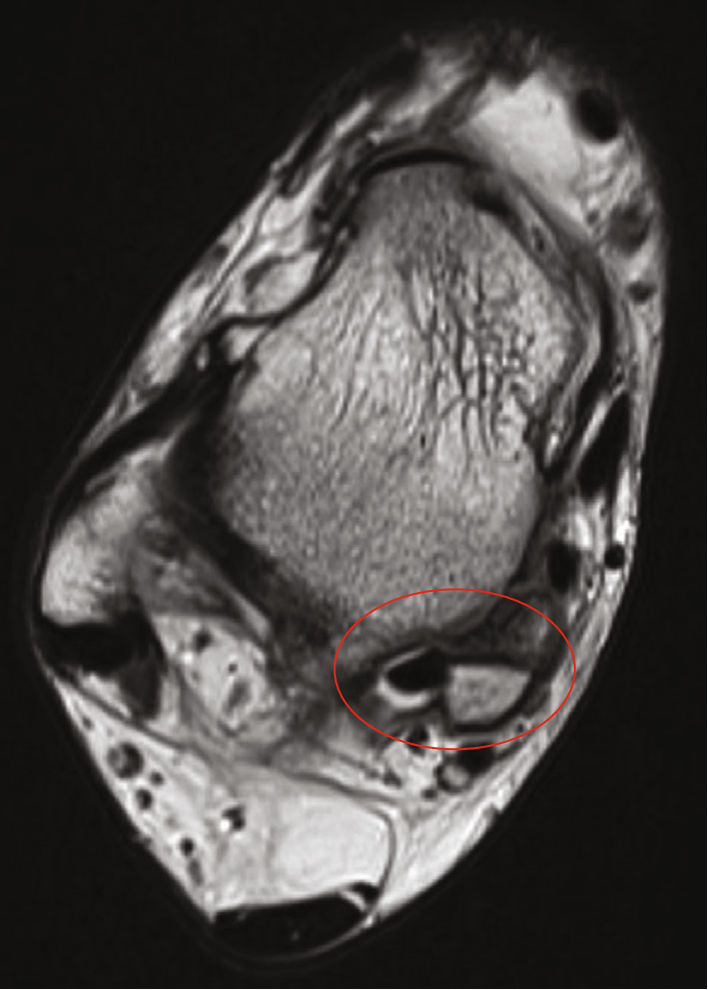
Axial slice of T2 MRI of right ankle demonstrating fluid enhancement around the FHL tendon with the adjacent fractured posteromedial talus fragment.

**Figure 4:**
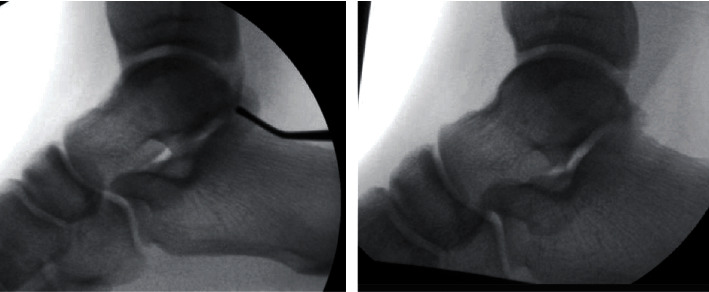
Intraoperative image intensifier imaging demonstrating pre- (left) and post- (right) excision of the posteromedial talus fragment.

A 32-year-old male architect was referred to the orthopaedic service with right PAIS. He described an injury to his right ankle several years ago as he went to kick the ball when playing football. He did not seek immediate medical attention, and the initial symptoms settled. He then went on to develop pain and medial-sided ankle swelling when he tried to carry out vigorous sporting activities some years later. On examination, there was a bony swelling behind his medial malleolus, and pain was exacerbated with plantar flexion and inversion of the ankle/foot demonstrating signs of PAIS. The MRI scans demonstrated a bony fragment with surrounding oedema associated with the medial talar tubercle. The fragment was between FDL and FHL tendons representing an “unusually located os trigonum,” and there was also associated FHL tenosynovitis (see Figure 3). Interestingly lateral X-rays did not show any abnormality as this patient did not have a conventional os trigonum. He underwent operative intervention with an open excision of the bony fragment (see Figure 4). The FHL tendon was found to have a split, and this was also repaired. The postoperative course was complicated with superficial wound infection which was successfully treated with a course of oral antibiotics. At the 6-week follow-up, the patient was pleased and described that their PAIS symptoms were improving.

### 2.3. Case 3

**Figure 5:**
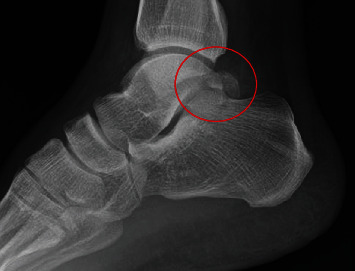
Lateral radiograph of right ankle demonstrating a prominent fractured and displaced posterior process of talus.

**Figure 6:**
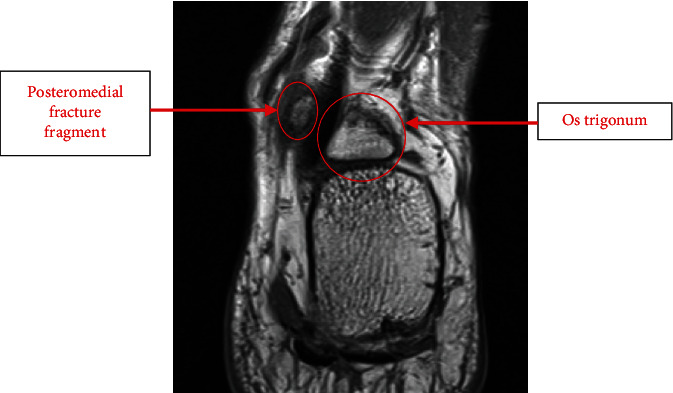
Coronal slice of T2 MRI demonstrating the large os trigonum lateral to the FHL tendon and the fracture fragment medial to the FHL tendon.

A 39-year-old male construction worker presented to the foot and ankle orthopaedic clinic with posteromedial left ankle pain following a minor football injury 6 months ago. He described a football tackle to his left ankle; subsequently, the ankle became swollen and painful. He did not seek immediate medical attention after the injury. Due to ongoing pain symptoms of PAIS, he was unable to carry on with sporting activities. On examination, his ankle was not swollen, and he had a palpable posteromedial lump that was tender. His pain film radiograph demonstrated a prominent posterior process of the talus that was fractured and displaced, but this had been misdiagnosed as an os trigonum (see Figure 5). He underwent an MRI to evaluate this lump and determine the relation to the medial neurovascular structures. The scan demonstrated a posteromedial tubercle fracture fragment medial to the FHL tendon. There was also an associated os trigonum. Interestingly, the MRI report did not describe the posteromedial tubercle fracture fragment that was the palpable mass (see Figure 6). This fragment was painful and restricted his mobility. He underwent operative intervention with open excision of the posteromedial fracture fragment and os trigonum. This was achieved through a posteromedial ankle incision where the neurovascular bundle and FHL tendon were seen and protected. He was followed up in 6 weeks where his PAIS symptoms had improved, and he was commenced on physiotherapy.

### 2.4. Case 4

**Figure 7:**
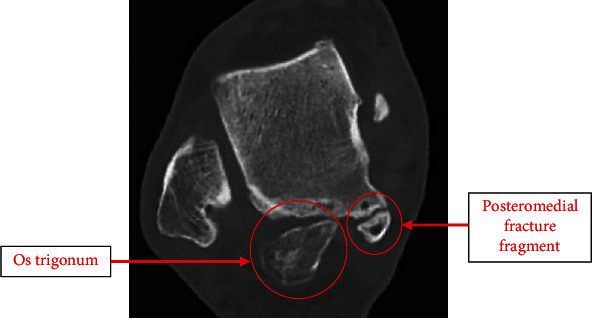
Axial CT slice demonstrating lateral os trigonum and posteromedial talus fracture fragment nonunion.

A 31-year-old male who works as a sports physiotherapist sustained a right ankle pronation injury whilst playing football. He did not seek immediate medical attention. He then presented 5 weeks later to the orthopaedic clinic complaining of deteriorating pain associated with ankle swelling and anterolateral ankle pain. He underwent an MRI that was reported with a small intra-articular effusion, a prominent os trigonum, lateral collateral ligamentous attenuation/fibrosis, and features of chronic posterior impingement. At the time, he was managed nonoperatively with a walking boot for 2 weeks and protected weight bearing. In 2018, he noted persistent posterior ankle pain. He did not sustain any new trauma. A right ankle CT scan demonstrated a malunion/partial union of the medial margin of the talus and secondary degenerative changes (see Figure 7). There was also still the presence of a prominent os trigonum with sclerotic changes associated with it. He was again managed nonoperatively with a walking boot and protected weight bearing during periods of exacerbation. He was able to return back to sporting activity once pain symptoms improved.

## 3. Discussion

PAIS secondary to an os trigonum or an excessively long Stieda's process arising from the lateral tubercle of the posterior talus is well understood and has been described in dancers and athletes including ballet, football, cricket, and runners [6]. It typically presents with posterior ankle pain on plantar flexion of the ankle; there may be a concomitant history of previous trauma. We describe a small subgroup of patients who present with PAIS secondary to impingement from delayed presentation of a fracture nonunion of the medial tubercle of the talus. Unlike the typical os trigonum, due to the oblique orientation of the talus, the medial tubercle lesions are not readily seen on the standard lateral X-ray projections (see Figures 1 and 5). Occasionally, a coexisting os trigonum may mislead clinicians as to the cause of the underlying impingement, and in others, a lack of an obvious abnormality may falsely reassure them. Awareness of this variant and its different clinical and radiological features is essential for a safe and successful treatment of this patient subgroup.

**Figure 8:**
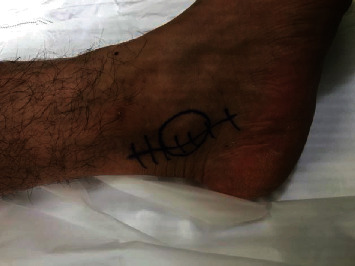
Medial palpable bony prominence marked with a circle and incision positioned posterior to the posterior tibial pulse.

We have noted that unlike os trigonum syndrome, PAIS secondary to the medial tubercle fracture presents with a subtle bony prominence inferior to the posterior edge of the medial malleolus, which becomes more appreciable only when compared to the contralateral normal ankle (see Figure 8). This can be tender to palpation or the patient may complain of pain in this location. Patients will also have positive posterior impingement signs and symptoms although the pain is especially worse with combined plantarflexion and inversion manoeuvers. Unlike conventional os trigonum syndrome, plain X-rays often fail to show the lesions which are readily visible on three-dimensional imaging. The bony fragments are often seen to be medial to the FHL tendon in contrast to the typical os trigonum which is on the lateral side of this tendon. This means that the medial tubercle fragments are closely related to the posterior tibial neurovascular bundle. For this reason, we advise against endoscopic excision of these fragments, a technique that is now routine for treating PAIS secondary to an os trigonum. We performed an open excision of the medial fragments on all patients protecting the neurovascular bundle under direct vision. If a symptomatic os trigonum was present, this was also excised through the same approach. All patients demonstrated improved symptoms of PAIS and were able to return back to sporting activity.

Fracture of the talar posterolateral tubercle is known as Shepard's fracture and can be mistaken radiographically for an os trigonum [7]. This fracture can be a result of forced hyperplantarflexion and inversion, causing compression of the posterolateral talus process between the calcaneum and the distal tibial rim [8]. A second mechanism described is an avulsion-type injury by the posterior talofibular ligament during hyperdorsiflexion and inversion [9]. Fracture of the talar posteromedial tubercle is known as Cedell's fracture, and it is described to be an avulsion-type fracture by the deltoid ligament during a pronation dorsiflexion injury [10]. This is the fracture type that was seen in all four cases described, and the fragment can also be mistaken for an unusually positioned os trigonum.

The aetiology of these lesions is therefore likely to be traumatic. All the patients in our series recalled previous “soft tissue” ankle injuries in relation to playing football. Interestingly, none had a diagnosis made at the time as they had not presented for medical attention with some not even recalling a significant single episode of trauma. This implies that these factures can occur without significant pain or swelling and may be underestimated by patients and emergency practitioners alike, only to present secondarily with symptoms of PAIS some time later. Football (soccer) was implicated in all of our patients.

Diagnosis of the os trigonum or talar fractures can be determined from a clinical history of PAIS and confirmatory radiography. Clinical suspicion of talar process fracture should govern obtaining further imaging. An oblique X-ray of the foot taken in external rotation has been useful to identify medial talus fractures that are not readily viewed on the lateral X-ray [11]. A CT scan can yields fractures that were not visualised on plain radiographs [12]. An MRI scan is useful if the osseous pathology cannot be visualised on plain radiography and is associated with surrounding posterior ankle soft tissue pathology suggesting chronic PAIS [13]. In the cases described, the MRI also revealed other soft tissue pathologies such as FHL tenosynovitis and lateral ligament sprain. All our cases had both the fracture fragments and an associated os trigonum, and in all cases, the os trigonum was initially incorrectly thought to be the cause of their PAIS.

Majority of talar process fractures presenting acutely can be managed nonoperatively with immobilisation and weight bearing restrictions. Displaced fractures can be managed with arthroscopic and/or open surgery to fix or excise the fracture fragment [3]. Treatment of PAIS related to os trigonum can be managed nonoperatively with activity modification, physical therapy, and corticosteroid injection with success in up to 84% of cases [14]. Nonoperative management was the plan for the last case we described. The patient was not experiencing pain that was debilitating, and it was not constant like the other cases. The patient was able to still carry out sporting activity between exacerbations. Thus, we opted for nonoperative management as symptoms were mild compared to the other cases.

Recalcitrant os trigonum syndrome can be managed surgically with various techniques described including endoscopic, arthroscopic, and open approaches [5]. The three cases that underwent surgical open excision of the fracture fragment were experiencing symptoms that were affecting their daily mobility. Arthroscopic surgery was not an option due to the risk of neurovascular injury.

### 3.1. Operative Technique

**Figure 9:**
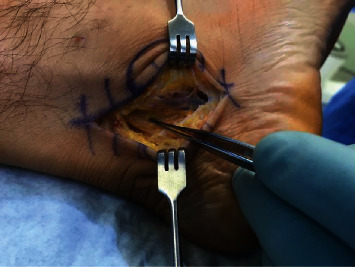
Neurovascular bundle pointed out with forceps.

**Figure 10:**
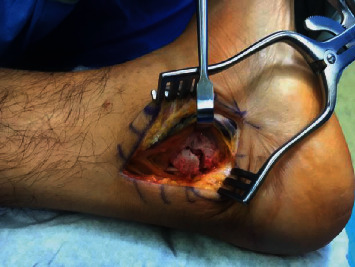
Posteromedial talus fracture nonunion fragment exposed below the neurovascular bundle on dorsiflexion maneuver.

Patients were positioned supine to access the medial ankle. A contralateral sandbag was used to improve access to the ipsilateral medial ankle. The posterior tibial pulse was palpated and marked. A well-padded high tourniquet was placed and inflated. The incision was over the bony lump posterior to the posterior tibial pulse (see Figure 8). The neurovascular bundle was seen and protected with blunt instrumentation/slings (see Figure 9). Dorsiflexion of the ankle can help clear the operative field of important neurovascular structures and exposed the fracture fragment (see Figure 10). The bony fragment was excised with the aid of osteotome impaction and bone nibblers. The FHL tendon lies lateral to the bony fragment and is inspected for any tears that can be repaired as a result of FHL tenosynovitis. If a symptomatic os trigonum coexisted, this was then excised at the same time. Tourniquet was deflated prior to closure to assess vascular structures and to achieve haemostasis. Postoperatively, patients are allowed to weight bear as pain allows using crutches. Bulky bandaging is used for the first two weeks to restrict ankle motion to allow soft tissue healing. Bandaging is removed, and physiotherapy commenced at the two-week point.

## 4. Summary Conclusion

- In patients presenting with PAIS but with a medially located bony prominence or posteromedially located pain, a posteromedial tubercle fracture nonunion should be suspected
- Plain lateral X-ray alone is not reliable; an oblique X-ray or three-dimensional imaging with CT or MRI is helpful to make an accurate diagnosis
- Care should be taken to differentiate it from a coexisting os trigonum
- The lack of a history of trauma should not deter the clinician from making a diagnosis of such fractures although previous minor “soft tissue” ankle injuries may be reported
- If surgery is considered, we advocate an open surgical excision of the medial tubercle and any coexisting os trigonum to avoid injury to the neurovascular bundle through endoscopic techniques

## Data Availability

No data was used to support this case series.
